# Indicators for Monitoring Water, Sanitation, and Hygiene: A Systematic Review of Indicator Selection Methods

**DOI:** 10.3390/ijerph13030333

**Published:** 2016-03-17

**Authors:** Stefanie Schwemlein, Ryan Cronk, Jamie Bartram

**Affiliations:** Department of Environmental Sciences and Engineering, University of North Carolina, Chapel Hill, NC 27599, USA; schwstef@gmail.com (S.S.); rcronk@live.unc.edu (R.C.)

**Keywords:** criteria, method, monitoring and evaluation, Sustainable Development Goals, WaSH, water

## Abstract

Monitoring water, sanitation, and hygiene (WaSH) is important to track progress, improve accountability, and demonstrate impacts of efforts to improve conditions and services, especially in low- and middle-income countries. Indicator selection methods enable robust monitoring of WaSH projects and conditions. However, selection methods are not always used and there are no commonly-used methods for selecting WaSH indicators. To address this gap, we conducted a systematic review of indicator selection methods used in WaSH-related fields. We present a summary of indicator selection methods for environment, international development, and water. We identified six methodological stages for selecting indicators for WaSH: define the purpose and scope; select a conceptual framework; search for candidate indicators; determine selection criteria; score indicators against criteria; and select a final suite of indicators. This summary of indicator selection methods provides a foundation for the critical assessment of existing methods. It can be used to inform future efforts to construct indicator sets in WaSH and related fields.

## 1. Introduction

Drinking water, sanitation, and hygiene (WaSH) are important for human health, well-being, and development [1]. In September 2015, the United Nations launched the Sustainable Development Goals (SDGs)—a set of 17 goals and 169 targets for sustainable human development. The SDGs include a goal for water and sanitation: to “ensure access to water and sanitation for all” by 2030 [2]. Since 1990, 2.6 billion people have gained access to improved water sources and 1.9 billion people have gained access to improved sanitation. However, 663 million people lack an improved water source and 2.4 billion people do not use improved sanitation [3]. 

Achieving the goal of water and sanitation for all and to reach the remaining unserved people will require more and better use of data. Important indicators, such as water system functionality, safe management of excreta, water quality, sustainability, sanitary risk, and the enabling environment, will need to be measured; and data will need to be disaggregated by gender, socioeconomic status, and disability status [3,4,5,6]. Improved monitoring and new indicators are needed in non-household settings such as health care facilities, workplaces, and schools [7,8]. 

Improved monitoring of WaSH conditions is needed to track progress, improve accountability, and demonstrate impact. Monitoring data can be used to inform policy development and resource investment. Monitoring data can be used to identify opportunities to adjust the implementation strategy of a project or program at an interim stage, thus contributing to improved results. 

Despite these benefits, stakeholders often do not allocate adequate resources to implement robust or sufficient WaSH monitoring [9]. Project and program monitoring do not always use consistent, specific, or relevant indicators. As a result, efforts to benchmark and report accurate data on the status of WaSH are limited. There are examples from the literature where analysis of WaSH monitoring data yielded important and valuable insights for policy and practice; but more value could have been derived if indicators were added or improved [10,11]. There is a considerable need to improve the quality and coordination of monitoring in order to identify weaknesses in data collection and to inform decisions in WaSH policy and practice [12].

An important component of effective WaSH monitoring is a framework for data collection based on a robust set of indicators. To improve quality and coordination of monitoring in WaSH, it is necessary to develop a structured process for data collection based on a set of indicators. Lorenz (2001) describes such an indicator set as “(an) aggregation of variables (that) describes a system or process such that it has significance beyond the face value of its components” [13]. Hammond *et al.* (2005) note two important characteristics of indicators that make them useful for decision-making: (1) they quantify information so its significance is more readily apparent; and (2) they simplify information about complex phenomena to improve communication [14]. Carefully constructed indicator sets may be useful for policymakers and other stakeholders because “they can provide valuable information on complex issues in a relatively accessible way” [15]. 

Selection methods for environment-related indicators are widely recognized to be “insufficiently systematic and transparent” [15]. The development and consistent use of objective and rigorous methods for indicator selection are needed for meaningful and credible WaSH monitoring. Reliable indicators facilitate comparison of projects, programs, and interventions.

In response to the need for WaSH monitoring improvements, this systematic review aims to identify methods for the selection of WaSH indicators. Existing indicator selection methods used in WaSH-related fields were reviewed. Based on the results of this review, a method for WaSH indicator selection is proposed. This review highlights indicator selection methods that can be applied to WaSH with the goal of promoting broader use and application of these methods in WaSH monitoring.

## 2. Methods 

### 2.1. Search Strategy 

Published, peer-reviewed literature was searched in October 2013 and May 2014 using the following electronic databases: PubMed; Web of Science; Global Health; and Academic Search Complete. The search was based on the keyword string: “indicator* AND selection AND criteria AND (environment* OR development OR public health OR water).” An expansive indicator selection literature exists beyond the field of WaSH; the authors recognize that a review of this literature could provide relevant insight to developing WaSH indicators. As such, literature from related fields was included, including environmental science and ecology, sustainability and international development, and water management. The body of indicator selection literature is vast, and the authors did not attempt to capture all of it in this review.

The search was restricted to papers for which there was an abstract and full-text article available in English. Papers were included in the review if they described methods used to identify, select and validate indicators relevant to the fields of environment, international development, and water. Studies analyzing clinical or biological indicators (e.g., fecal indicators, biomarkers, human samples) were excluded, as they are beyond the scope of this paper. Dissertations and non-peer reviewed (*i.e.*, “grey”) literature were not included in the review. The search had no restrictions on time or location. 

After the initial electronic database search was conducted, reference titles were screened, and an abstract screening was conducted. Full-text articles of selected references were reviewed, and articles that passed inclusion and exclusion criteria were included for analysis.

### 2.2. Data Extracted from Literature

Each paper was reviewed for use of distinct indicator selection methods. After the indicator selection methods were identified, a second full text review was performed to tally every method that each article used or proposed for indicator selection. A six-stage method of indicator selection was developed based on perceived relevance and the frequency with which each method was tallied in included studies.

## 3. Results 

### 3.1. Systematic Literature Review

#### 3.1.1. Search Results

**Figure 1:**
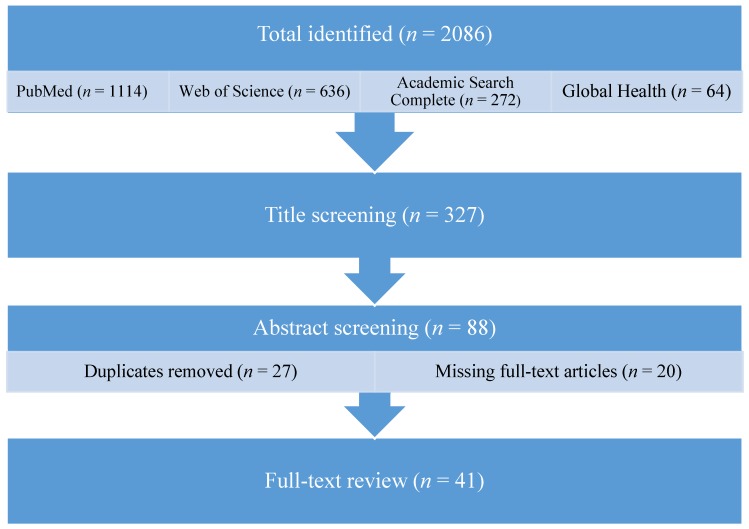
Literature search flow diagram. *n* indicates the number of studies remaining after each identification or review stage.

The primary literature search yielded 2086 references, with the majority of results from PubMed (53%). Title screening yielded 327 potentially relevant results. A secondary screening of abstracts yielded 152 potentially relevant papers; 88 remained after removing duplicates (27 articles) and references for which the full-text article was not available online (20 articles). Forty-one references were included after the final, full-text screening (Figure 1). 

#### 3.1.2. Classification of Studies Included in Review

Of the 41 papers included in the review, 20 were in the fields of ecology and environment (49%); 12 papers addressed issues of sustainability and/or international development (29%); and the remaining nine were specific to water management and/or WaSH (22%) (Table 1).

The included studies aimed to measure a variety of complex concepts and used different conceptual frameworks to organize indicator sets. Ostrom notes that one major aim of a conceptual framework is to “identify the universal elements that any theory relevant to the same kind of phenomena would need to include” [16]. Hammond *et al.* (2005) observe that data from a large set of indicators can be diverse and confusing; as such, a framework is needed to structure information “to make it more accessible and intelligible to decision-makers and the general public” [14]. Ostrom further notes that the role of frameworks is to identify individual indicators and determine “general *relationships among these elements* that one needs to consider for institutional analysis” (emphasis added) [14].

Framework types frequently used to organize indicator sets include the Driving forces-Pressures-State-Impact-Response (DPSIR) (*n* = 5), Social-Economic-Ecologic/Environment (SEE) (*n* = 11), and Pressure-State-Response (PSR) (*n* = 2). The SEE framework is often used to describe foundational considerations for sustainability in business [17]. DPSIR and PSR are both causal chain frameworks. Causal chain frameworks are described by Neimeijer and deGroot (2005):

“In the causal chain, social and economic developments are considered driving forces that exert pressure on the environment, leading to changes in the state of the environment. In turn, these changes lead to impacts on human health, ecological systems and materials that may elicit a societal response that feeds back on the driving forces, pressures, or on the state or impacts directly.”

[15]

In addition to these three framework types, 16 studies developed frameworks specific to the factor or a concept that the indicator set aimed to measure (factor specific, FS). Six studies did not use or recommend using a framework for structuring indicators (15%). A catalog of all studies included in the review and their respective fields of study are included in Table 1. 

Studies included that focused on WaSH or water management used diverse types of criteria to select indicators (these studies are listed in Table 1). Authors relied on consulting experts or stakeholders (whether through formal Delphi techniques or informal facilitation methods), multi-criteria analysis, or selecting indicators on an *ad hoc* basis [18,19,20,21,22,23]. Others emphasize the importance of transparency in the process and continuous assessment of the indicators throughout their lifecycle of use in monitoring [13,24]. 

Ten methodological stages that emerged from the review were cataloged: (1) constructing a theoretical or conceptual framework (*n* = 33); (2) conducting a literature review to find the initial list of indicators (*n* = 22); (3) defining the purpose of the indicator set (*n* = 31); (4) determining selection criteria (*n* = 33); (5) weighting selection criteria (*n* = 8); (6) evaluating individual indicators (*n* = 29); (7) evaluating a set of indicators (*n* = 8); (8) consulting stakeholders (*n* = 27); (9) final indicator selection (*n* = 33); (10) applying methods to a case study (*n* = 24). The highest number of methodological stages used in a single paper was nine [29,32,45], and the lowest was four [20,39,41,49]. A description of each methodological stage is provided in Table 2, and a tally of every method that each article used or proposed for indicator selection is included in Table 3.

### 3.2. Proposed Method for Selecting Indicators for WaSH Monitoring

**Figure 2:**
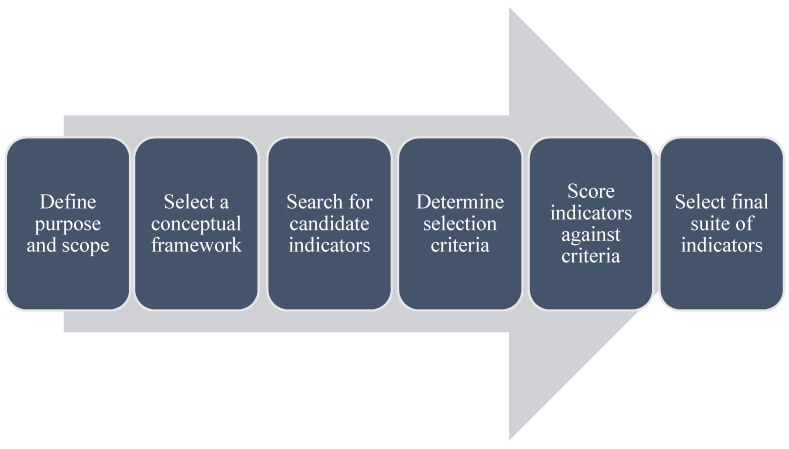
Proposed method for selection of indicators for WaSH monitoring.

Using the systematic review results (Section 3.1), a six step method for selecting WaSH indicators is proposed. The six steps are selected because of their frequency of use in studies included in the systematic review and their applicability to WaSH monitoring (Figure 2). 

#### 3.2.1. Define Purpose and Scope 

To produce a useful and meaningful set of indicators, it is necessary to define the concept of interest for monitoring and establish the purpose and scope of the indicator set [15]. This stage helps to frame the subsequent steps by establishing a precisely defined set of goals. The concept and purposes may be broadly or narrowly defined, depending on user needs and the intended audience. A possible example of a purpose and scope of indicators applied to WaSH is “to identify a set of elements that reflect the status of WaSH in school settings on the national level.”

#### 3.2.2. Select a Conceptual Framework

The purpose of a conceptual framework is to provide organizational structure for categorizing and combining indicators in a logical and useful way [58]. Existing frameworks that could possibly function for WaSH indicator sets include Driving forces-Pressures-State-Impact-Response (DPSIR), Social-Economic-Ecologic/Environment (SEE), and Inputs-Outputs-Outcomes-Impacts.

#### 3.2.3. Search for Candidate Indicators

Candidate indicators are extracted through a literature review of existing WaSH indicators and other relevant WaSH literature. New indicators can also be proposed during this stage if deemed appropriate by researchers or policy-makers. 

#### 3.2.4. Determine the Selection Criteria

Candidate indicators should be evaluated using indicator selection criteria. Examples of indicator selection criteria include:

- Measurable: Indicator is straightforward to measure and quantifiable [59]
- Reliable: Indicator measurement produces the same value if repeated in the same way on the same population at almost the same time [60]
- Data available: Data are available and accessible, accurate, comparable over time, complete with historical information and covering sufficient geographic areas [57]
- Sensitive: Indicator reveals important changes in the factor of interest [60]
- Valid: There must be an accurate correlation between an indicator and the issue for which it is supposed to proxy [60]

#### 3.2.5. Score Indicators against Criteria

The adopted selection criteria can be used to screen candidate indicators. Each candidate indicator is scored for its suitability in relation to each criterion. The indicator is assigned a score of 0 if the indicator does not meet the selection criterion, or 1 if the indicator does meet the selection criterion. A score of 0.5 is assigned if the indicator meets the selection criteria in some instances and not in others. 

#### 3.2.6. Select Final Set of Indicators

Based on the indicator scoring results, a final set of indicators is chosen. In order to obtain a final set that includes a relatively even spread of indicators across each step of the selected framework, only indicators that receive scores at least one standard deviation above the mean within each category can be selected.

## 4. Discussion

In this study, we reviewed methods of indicator selection and identified six methodological stages for selecting indicators for WaSH. Indicator selection methods are not always used in WaSH which is one of the reasons why this review was conducted. We present a summary of indicator selection methods for environment, international development, and water. This review provides a foundation for critical assessment of indicator selection methods and can be used to inform future efforts to construct indicator sets in WaSH.

The findings display considerable homogeneity in methods for indicator selection across fields of study, providing a foundation for the construction of commonly used methods for indicator selection. This homogeneity in indicator selection methods is evident in two ways: the relatively small number of distinct methods identified (ten methods were identified across 41 studies); and the frequency with which each method was used across the studies (eight of the methods were used by over half of the studies). 

Though homogeneity and frequency of use are not necessarily indicative of good practices or appropriateness for the purposes of this study, it does provide some validation of the selected methods, as it suggests that other researchers have found these methods to be useful. The methods provide logical organization to the indicator selection process, eliminating the arbitrariness often associated with indicator selection. Additionally, the defined process improves transparency, allowing other researchers or stakeholders to better understand each stage in the decision-making process. 

Indicators that can be used across studies, projects, and interventions are needed in WaSH. Clear, consistent indicators in WaSH can be used to make comparisons in status over space and time. They can be used to describe complex implementation and they help to provide an overview of projects and programs. Data collected from indicators can be used to identify trends and patterns and can be used to make improvements over time. Data can be used in making important policy and practice decisions. Comparable indicators can be used to measure performance of projects, programs or countries [61]. In contrast, poor indicators may produce data that lead to an incorrect understanding of a project or program. 

However, indicators can have limitations. For example, data collected through surveys are collected only at a point in time and represent a “snapshot” of the situation whereas the status of, for example a water system, may change seasonally [62]. Indicators do not necessarily reveal the entire situation of a project or program and data must be interpreted with care. 

A limitation of this study was the exclusion of grey literature, and there are useful resources available [63]. We only reviewed English language publications which is another limitation. This study revealed a number of areas for future research. Further analysis is needed to determine how to evaluate interrelations between indicators. Additional methodological refinement is needed to determine the usefulness of a weighting scheme for selection criteria. Few studies comment on the strengths and weaknesses of their indicator selection criteria. Assessment of strengths and weaknesses would be valuable to determine which methods are most useful when selecting indicators for WaSH projects and programs. 

Further work is needed to determine appropriate scale and weights for consolidation of the indicators into a composite index. A composite index is a compilation of individual indicators into a single value on the basis of an underlying model [57]. Such composite indices are controversial because of their tendency to mask important aspects of complex concepts; however, such an index, if constructed properly, can serve as an important tool to facilitate monitoring and interpreting general trends in WaSH conditions globally. Finally, more research is needed to determine how a set of indicators can better monitor and reflect needs on scales ranging from household and community level to national and global levels.

## 5. Conclusions

This review highlights indicator selection methods that can be applied to WaSH monitoring. The review fills a knowledge gap since many WaSH monitoring initiatives do not always use well designed indicators. This review may help promote broader use and application of these methods in WaSH monitoring. 

## Figures and Tables

**Table 1:** Studies included in the review, grouped by field of study.

Reference	Factor Measured ^a^	Framework Type ^b,c^
**Ecology and Environment**		
Breckenridge *et al.* (1995) [25]	Ecological conditions of range-lands	FS
Dinsdale and Harriott (2004) [26]	Anchor damage; coral reef health	FS
Doren *et al.* (2009) [27]	Ecosystem health	Mod DPSIR
Fontalvo-Herazo *et al.* (2007) [28]	Marine and coastal system health	FS
Gomontean *et al.* (2008) [29]	Forest ecosystem health	FS
Greene and Tonjes (2014) [30]	Environmental benefits of municipal waste systems	FS
Lebacq *et al.* (2013) [31]	Sustainability of livestock systems	Mod SEE
Maes *et al.* (2011) [32]	Effects of forest management	FS
Malecki *et al.* (2008) [33]	Environmental public health surveillance system capacity	-
Mangoyana *et al.* (2013) [34]	Sustainability of biofuel systems	FS
Monroy-Ortiz *et al.* (2009) [35]	Importance of plant species to local conservation	FS
Niemeijer and de Groot (2008) [15]	State of the environment	Mod DPSIR
Puig *et al.* (2014) [36]	Sustainable port development	FS
Rice and Rochet (2005) [37]	Ecosystem effects of fishing	-
Rodriguez-Piñeros and Lewis (2013) [38]	Sustainable forest management	FS
Rubio and Bochet (1998) [39]	Desertification risk	-
van Oudenhoven *et al.* (2012) [40]	Effects of land management on ecosystem services	Mod DPSIR
Zalidis *et al.* (2004) [41]	European Union agri-environmental measures effectiveness	DPSIR
Zhen and Routray (2003) [42]	Agricultural sustainability in developing countries	SEE
Zucca *et al.* (2012) [43]	Land degradation and desertification	Mod DPSIR
**Sustainability and International Development**		
Afgan *et al.* (2000) [44]	Sustainability of energy systems	Mod SEE
Bobbitt *et al.* (2005) [45]	Well-being, public health	FS
Buchholz *et al.* (2009) [46]	Sustainability of energy systems	SEE
Castillo and Pitfield (2010) [47]	Sustainability of transport systems	-
Corbière-Nicolliera *et al.* (2011) [48]	Sustainability of energy systems	SEE
Doody *et al.* (2009) [49]	Sustainable development	-
Joumard *et al.* (2011) [50]	Environmental impact (transport sector)	Mod PSR
Kurka (2013) [51]	Regional sustainability of bioenergy developments	Mod SEE
Kurka and Blackwood (2013) [52]	Sustainability of energy systems	Mod SEE
Lin and Lu (2013) [53]	Ecotourism sustainability	Mod SEE
Liu (2014) [54]	Sustainability of renewable energy systems	SEE
Tanguay *et al.* (2013) [55]	Sustainability of tourism industries	SEE
**Water Management and WaSH**		
Cools *et al.* (2013) [18]	Wetland management	FS
Garfi and Ferrer-Marti (2011) [19]	Conditions of rural water and sanitation projects in developing countries	Mod SEE
Garriga and Foguet (2010) [20]	Water stress and scarcity	FS
James *et al.* (2012) [24]	Water quantity	FS
Juwana *et al.* (2010 a, b) [21,22]	Sustainability of water resources	FS
Kim and Chung (2013) [23]	Vulnerability of water supply to climate change	FS
Lorenz *et al.* (2001) [13]	Pressures on river ecosystems	Mod PSR
Singhirunnusorn and Stenstrom (2009) [56]	Appropriateness of wastewater treatment systems	-

**^a^** “Factor measured” refers to the issue or topic that the indicators in the study aim to measure; **^b^** “Framework type” refers to the organizational model used to structure the indicator set. The specific frame- work details were extracted then categorized by the types that were found; **^c^** “FS” = factor-specific = study developed a framework specific to the factor or concept being measured; “Mod” = modified version of framework type listed; “DPSIR” = Driving forces-Pressures-State-Impact-Response; “SEE” = Social-Economic-Environmental/Ecologic; “PSR” = Pressure-State-Response; “-” = framework not provided in the study.

**Table 2:** Definitions of methods cataloged from indicator selection literature.

Method Used in Literature	Definition
Theoretical/conceptual framework	Organizational structure to categorize indicators; provides the basis for the selection and combination of variables into a meaningful composite under a fit-for-purpose principle [57]
Literature review for initial indicators	Preliminary list of indicators is constructed following a thorough literature review of existing indicators for the concept in question
Defining the purpose of the indicator set	The concept being measured by the indicator suite is explicitly defined
Determining selection criteria	A list of quality criteria by which the initial list of indicators should be screened is defined
Weighting selection criteria	Selection criteria are rated or ranked into a weighting scheme (either qualitative or quantitative) that reflects the importance of each criterion
Evaluating individual indicators	Each initial indicator is scored based on the extent to which it meets the defined selection criteria
Evaluating set of indicators	Full set of indicators is scored based on the extent to which it accurately and holistically represents the concept being measured
Consulting stakeholders	Experts or other stakeholders in the field of study are consulted for input on appropriateness of indicators, frameworks, and/or methods used
Final selection	Based on results from the criteria screening, stakeholder/ expert feedback, or some other criteria, a final set of indicators is selected from the initial list
Case study	Indicator selection methods are applied to select a set of indicators, and then data for each indicator is collected

**Table 3:** Catalog of indicator selection methods used in included literature.

Reference	Theoretical/Conceptual Framework	Lit Review for Initial Indicators	Defining Purpose of Indicators	Determining Selection Criteria	Weighting Selection Criteria	Evaluating Individual Indicators	Evaluating Set of Indicators	Consulting Stakeholders	Final Selection	Case Study
**Ecology and Environment**										
Breckenridge *et al.* (1995) [25]	X		X	X	X	X		X	X	X
Dinsdale and Harriet (2004) [26]	X	X	X	X		X			X	X
Doren *et al.* (2009) [27]	X	X	X	X		X		X	X	X
Fontalvo-Herazo *et al.* (2007) [28]	X		X				X	X	X	X
Gomontean *et al.* (2008) [29]	X	X	X	X	X	X		X	X	X
Greene and Tonjes (2014) [30]	X	X		X		X				X
Lebacq *et al.* (2013) [31]	X	X	X	X		X	X	X	X	X
Maes *et al.* (2011) [32]	X	X	X	X	X	X		X	X	X
Malecki *et al.* (2008) [33]		X	X	X		X				X
Mangoyana *et al.* (2013) [34]	X		X					X	X	X
Monroy-Ortiz *et al.* (2009) [35]										
Niemeijer and de Groot (2008) [15]	X		X	X		X	X		X	
Puig *et al.* (2014) [36]	X	X	X	X		X		X	X	X
Rice and Rochet (2005) [37]			X	X	X	X		X	X	
Rodriguez-Piñeros and Lewis (2013) [38]	X	X				X		X		X
Rubio and Bochet (1998) [39]			X	X		X			X	
van Oudenhoven *et al.* (2012) [40]	X	X	X	X		X		X	X	X
Zalidis *et al.* (2004) [41]	X		X	X					X	
Zhen and Routray (2003) [42]	X	X	X	X					X	
Zucca *et al.* (2012) [43]	X	X	X	X		X	X	X	X	X
**Sustainability and International Development**										
Afgan *et al.* (2000) [44]	X		X		X					
Bobbit *et al.* (2005) [45]	X	X	X	X		X		X	X	X
Buchholz *et al.* (2009) [46]	X	X		X		X		X		
Castillo and Pitfield (2010) [47]		X	X	X	X	X	X	X	X	
Corbière-Nicolliera *et al.* (2011) [48]	X		X	X		X		X	X	X
Doody *et al.* (2009) [49]				X		X		X	X	
Joumard *et al.* (2011) [50]	X	X	X	X		X		X	X	
Kurka (2013) [51]	X		X	X	X	X	X	X	X	X
Kurka and Blackwood (2013) [52]	X	X		X		X		X	X	
Lin and Lu (2013) [53]	X	X	X					X	X	X
Liu (2014) [54]	X		X	X	X	X			X	
Tanguay *et al.* (2013) [55]	X	X	X	X		X		X	X	
**Water Management and WaSH**										
Cools *et al.* (2013) [18]	X		X			X		X	X	X
Garfi and Ferrer-Marti (2011) [19]	X	X	X	X		X			X	
Garriga and Foguet (2010) [20]	X			X					X	X
James *et al.* (2012) [24]	X	X	X	X	X	X	X		X	X
Juwana *et al.* (2010 a, b) [21,22]	X	X		X				X	X	X
Kim and Chung (2013) [23]	X			X	X		X	X	X	X
Lorenz *et al.* (2001) [13]	X		X	X				X		
Singhirunnusorn and Stenstrom (2009) [56]			X	X	X	X		X	X	X
